# Effective energy harvesting from a single electrode based triboelectric nanogenerator

**DOI:** 10.1038/srep38835

**Published:** 2016-12-13

**Authors:** Navjot Kaur, Jitendra Bahadur, Vinay Panwar, Pushpendra Singh, Keerti Rathi, Kaushik Pal

**Affiliations:** 1 Centre of Nanotechnology, Indian Institute of Technology Roorkee, Roorkee-247667, India; 2 Department of Mechanical and Industrial Engineering, Indian Institute of Technology Roorkee, Roorkee-247667, India

## Abstract

The arch-shaped single electrode based triboelectric nanogenerator (TENG) is fabricated using thin film of reduced graphene oxide nanoribbons (rGONRs) with polyvinylidene fluoride (PVDF) polymer used as binder to effectively convert mechanical energy into electrical energy. The incorporation of rGONRs in PVDF polymer enhances average surface roughness of rGONRs/PVDF thin film. With the combination of the enhancement of average roughness and production of functional groups, which indicate improve charge storage capacity of prepared film. Furthermore, the redox peaks obtained through cyclic voltammetry were identified more in rGONRs/PVDF composite in comparison to pristine rGONRs to confirm charge transfer capability of film. Herein, the output performance was discussed experimentally as well as theoretically, maximum voltage was obtained to be 0.35 V. The newly designed TENG to harvest mechanical energy and opens up many new avenues of research in the energy harvesting applications.

Nowadays, energy harvesting is an area of immense interest because of the increasing energy demands of society to create self-powered and autonomous systems^1^. Energy harvesting from ambient using nanotechnology is an efficient approach to promote nanodevices, these consume very less amount of energy^2^. Nanogenerators has been categorized on the bases of pyroelectric^3^,^4^,^5^, piezoelectric^6^,^7^,^8^ and triboelectric^9^,^10^ effects, which are used to collect energy from the environment and convert it into electrical energy. Among the various energy harvesting techniques, triboelectric effect is one of the efficient phenomenon in our day to day life that a material develops electrical charges when it comes in contact with a dissimilar material through friction or by applying load i.e. mechanical energy^9^.

Dr. Wang and his group in 2012 at Georgia Institute of Technology devised a new-fangled means of generating electricity from mechanical energy based on triboelectrification and electrostatic induction^10^. The triboelectric nanogenerator (TENGs)^10^,^11^,^12^,^13^,^14^,^15^,^16^,^17^,^18^,^19^,^20^,^21^,^22^,^23^,^24^,^25^,^26^,^27^,^28^,^29^,^30^,^31^,^32^,^33^ are based on the phenomenon of electrostatic induction and triboelectric effect. These nanogenerators are found to be reliable, flexible, worthwhile and highly competent device to harvest mechanical energy into electrical energy. TENGs have fascinated much more attention due to their high energy conversion efficiency and high output. TENGs have wide areas of applications in portable electronic, tracking system, however an external source is essential to drive these sensors. The materials would produce potential drop, due to positive and negative polarity of the materials and would drive electrons to drift through an external load and give continuous outputs. The principle of TENG including four modes of operation such as: vertical contact separation mode^11^,^12^,^13^,^14^, free standing triboelectric mode^16^, sliding mode^17^,^18^,^19^,^20^,^21^,^22^,^23^ and single electrode mode^24^,^25^,^26^. The basic structure of all TENGs comprises two electrodes, which are essential for them to function. Within the past five years, many research groups have been demonstrated the TENG used both the electrodes of polymers^10^,^11^,^17^,^22^, polymers, metals^12^,^14^,^15^,^18^,^19^,^23^,^24^,^25^ and carbon based nanofillers such as graphene oxide (GO) and graphene^27^,^28^,^29^. Tian *et al*. and his co-workers have been fabricated the flexible nanogenerator based on graphene oxide (GO) thin film^27^. S.-W. Kim and his group have been fabricated the flexible and transparent nanogenerator^28^ based on graphene 1-layer (L), 2L, 3L and 4L. Multiple research groups have been developed different architectures contain single electrode with contact separation mode. Yang *et al*.^24^ has also been reported TENG based on single electrode. The quickly advance TENG have been predicted as highly capable, cost effective and next big thing for harvesting sundry forms of mechanical energy. To the best of our knowledge till to date no one has tried the rGONRs/PVDF as an effective corporal in devices and systems, those harvest energy.

rGONRs have interesting properties and unique structure^34^, which exhibited a high length to diameter ratio, straight edges and have amount of oxygen containing functional groups^35^,^36^, which could be enhance the negative charge on the surface of rGONRs, because oxygen is highly electronegative. In addition, edged structure can also offer a plenty of space for chemical modification^35^. Due to these unique properties, they are used in various other applications including supercapacitors^37^,^38^, as a hole transporting material in solar cells^39^,^40^, field effect transistors (FETs)^41^ and biomedicals etc.^36^,^38^.

In this communication, we ripen a strategy pointing to construct TENG with one of its electrode of composite material which contains oxidized graphene nanoribbons and PVDF as binder. Mainly, our approach focus on efficient way to improve the performance of TENG.

## Results and Discussion

**Figure 1:**
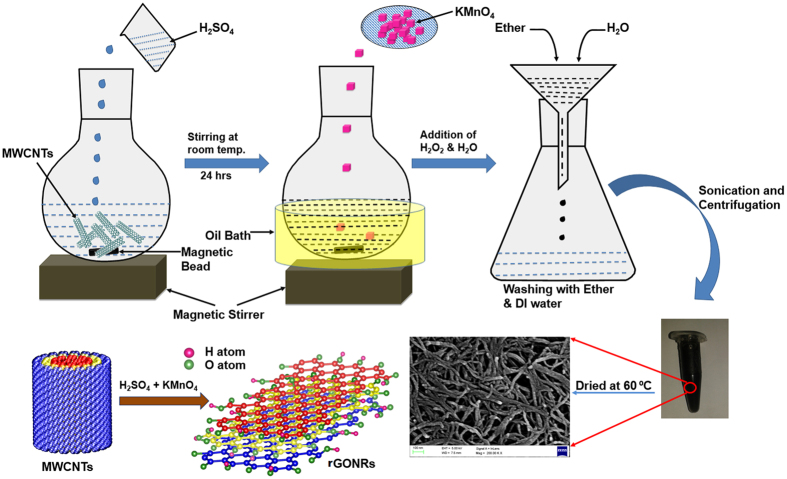
Schematic diagram of synthesis process for rGONRs from unzipping of CNTs through chemical treatment.

rGONRs were synthesized as discussed in the methods and schematic diagram of the synthesis process was illustrated in Fig. 1.

### Characterization of rGONRs

**Figure 2:**
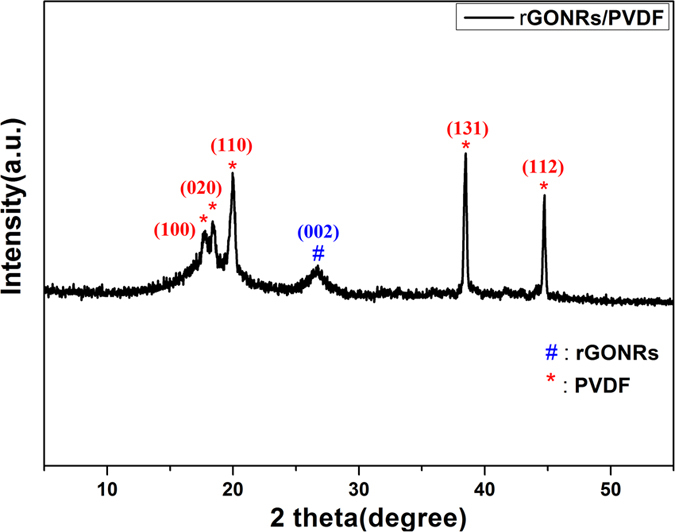
X-ray Diffraction (XRD) analysis of the rGONR/PVDF thin film.

X-ray diffraction (XRD) analysis was performed for the thin film prepared by rGONRs/PVDF composite as illustrated in Fig. 2. Figure 2 reveals that the characteristic peaks of PVDF polymer are present around 17.2°, 18.3°, 19.6°, 38.5° and 44.69° which are in good agreement with the reported literature [JCPDS No. 42-1650]. The peak around 26.5° confirms the presence of highly reduced graphene oxide nanoribbons in composites. In addition, the existence of all individual peaks of both elements indicates that they exists in their original phases and there is no direct interaction which changes the phase of overall product. Therefore, we have considered the combination of these elements as a rGONRs/PVDF composite material.

**Figure 3:**
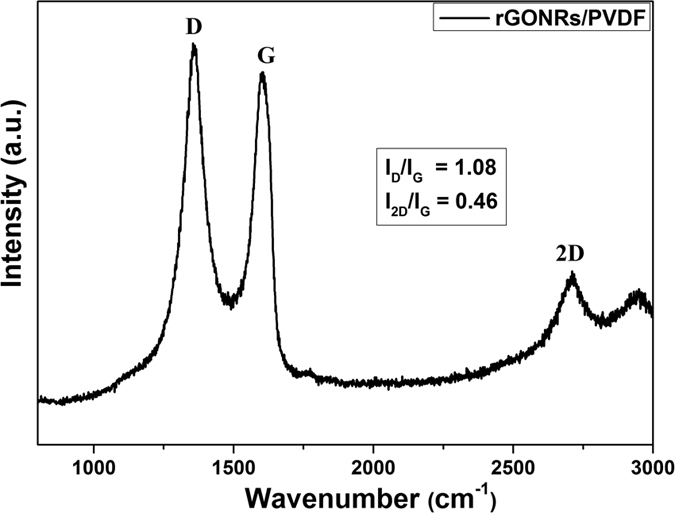
Raman spectra of rGONRs/PVDF thin film.

Raman spectroscopy of rGONRs/PVDF composite has been shown in Fig. 3. The presence of D-band at around 1360 cm^−1^ proves the presence of certain defects in the structures of rGONRs. These defects must be due to the presence of PVDF or some vacancies in the hexagonal structure created during opening of CNTs. Further, the observed value of I_2D_/I_G_ from the Raman spectrum indicates the presence of 2–3 layers stacked together^42^.

**Figure 4:**
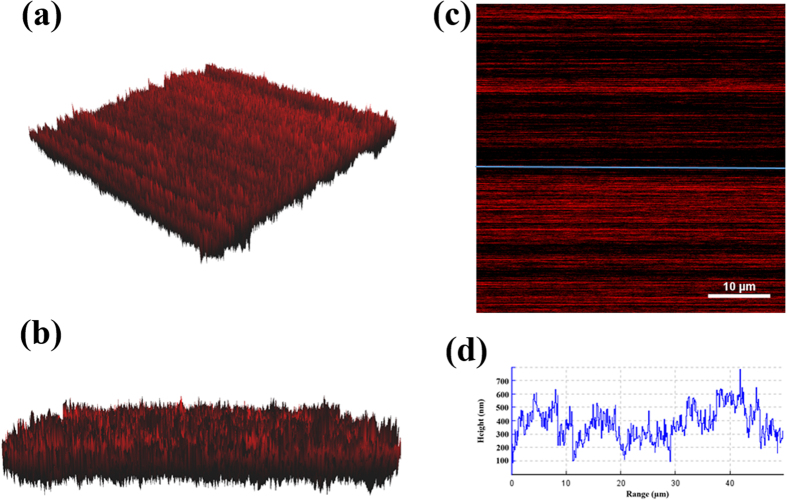
3D-AFM image of the rGONRs/PVDF thin film (**a** & **b**) Side view. (**c**) Top view. (**d**) Height profile of thin film.

Atomic Force Microscopy (AFM) images of rGONRs/PVDF thin film was shown in Fig. 4. In this analysis, we can observe the orientation and 3D topographical information about the sample surface. Figure S1 in the Supplementary Information shows the surface profilometry of the pristine rGONRs thin film as well as the rGONRs/PVDF thin film. From Figure S1, it has been clearly observed that, average surface roughness of rGONRs/PVDF thin film higher than rGONRs film. The average surface roughness play vital role to trap charge and enhance charge storage capacity of materials.

The degree of oxidation of graphene nanoribbons from MWCNTs were further illustrated by Fourier Transformation Infrared (FTIR) Spectroscopy. FTIR spectroscopy (Figure S2, Supplementary Information) shows that the −OH stretching was present at 3441.72 and 2923.43 cm^−1^ in the high frequency range, −CH stretching and carboxylic group (−COOH) at 2358.12 cm^−1^ and 1634.23 cm^−1^, respectively. Moreover, −CO stretching would be verified at 1384.05, 1270.20 and 1091.81 cm^−1^.

To extend our investigation, we had also examined the contact resistance by using two - probe resistivity measurement method. Figure S3 shows the current voltage (I-V) curve in which the contact resistance for pristine rGONRs have been found 6.11 Ω/μm^2^. While, composite was prepared using PVDF as binder, contact resistance increases upto 9.32 Ω/μm^2^ because of the presence of functional groups in rGONRs/PVDF. The Cyclic Voltammetry (CV) analysis was performed to examine electrochemical performance of the electrodes. CV curve (Figure S4 in Supplementary information) of prepared sample measured at scan rate of 100 mV/s in 1M DMF with in potential range from −1.5 to +1.5 V. The redox peaks were identified more in rGONRs/PVDF composite in comparison to the pristine rGONRs, which confirm better charge transport of rGONRs/PVDF composite. Moreover, area under the CV curve of rGONRs/PVDF is larger than the pristine rGONRs, so that the charge storage capability of this material is good in comparison to pristine rGONRs, this results are in good agreement with the literature^43^.

**Figure 5:**
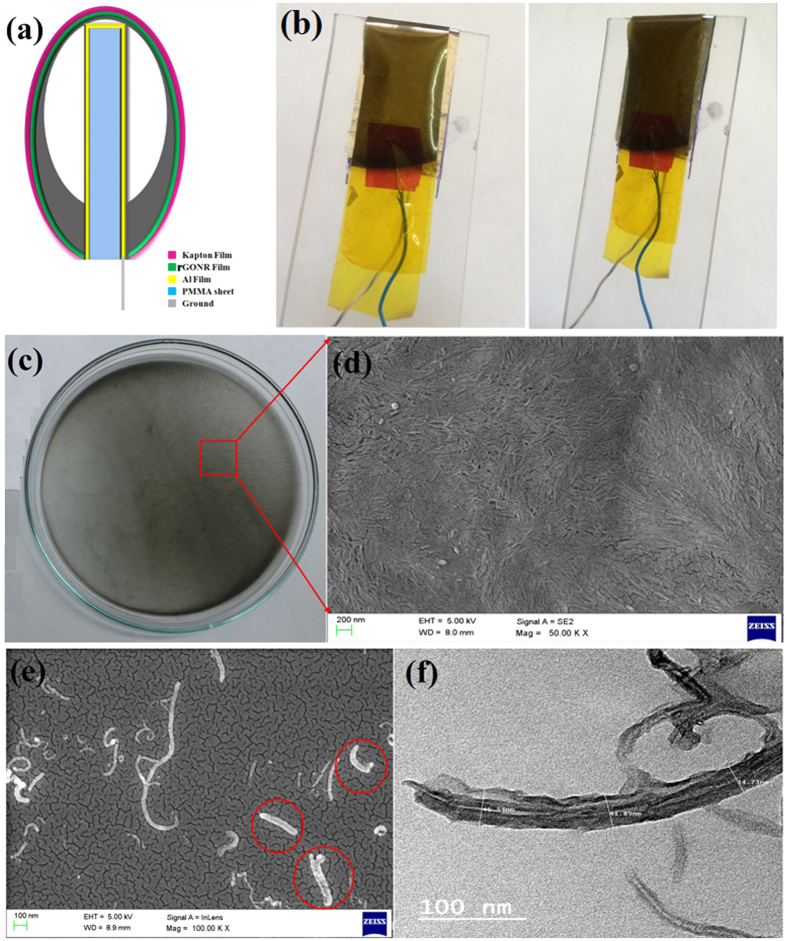
(**a**) Schematic view of the rGONRs/PVDF based triboelectric nanogenerator. (**b**) Optical images of triboelectric nanogenerator. (**c**) Thin film of rGONRs with PVDF. (**d**) FE-SEM image of thin film of rGONRs/PVDF composite. (**e**) FE-SEM image of rGONRs. (**f**) TEM image of rGONRs.

Field Emission-Scanning Electron Microscope (FE-SEM) and Transmission Electron Microscope (TEM) images of rGONRs are shown in Fig. 5e and f. These figures demonstrated that CNTs were unzipped completely in the form of oxidized graphene nanoribbons, having *~*45 nm width. The elemental analysis is obtained using energy dispersive X-ray analysis (EDAX) of the prepared rGONRs, which confirms the composition by weight% of carbon and oxygen are found 66.20% and 33.80%, respectively (Figure S5).

Figure 5b shows the optical images of fabricated rGONRs/PVDF based TENG, where one electrode is made ground and from the other we get voltage. These optical images clearly show the arch-shaped structure of TENG. The surface of thin film with uniformly distributed nanoribbons which are clearly visible in the FE-SEM image of thin film as shown in Fig. 5d. The obtained rough surface of the thin film would enhance the surface area of the device and due to this the device would give effective output^44^.

The rGONRs should be negatively charged because graphene oxide is negative charged as reported^45^. The charge on the rGONRs/PVDF film is fixed and confined by kapton tape. The total negative charge (Qr_GONRs_) of rGONRs/PVDF film could induce positive charge in the Al foil. The fabricated device as flat panel capacitor, the capacitance (C) of capacitor could be written as C = εS/d, where ε, S, d and represents dielectric constant of air, surface area of rGONRs/PVDF film and separation distance between rGONRs/PVDF film and Al foil. The output current and voltage could be based on relation as:


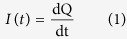



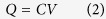


**Figure 6:**
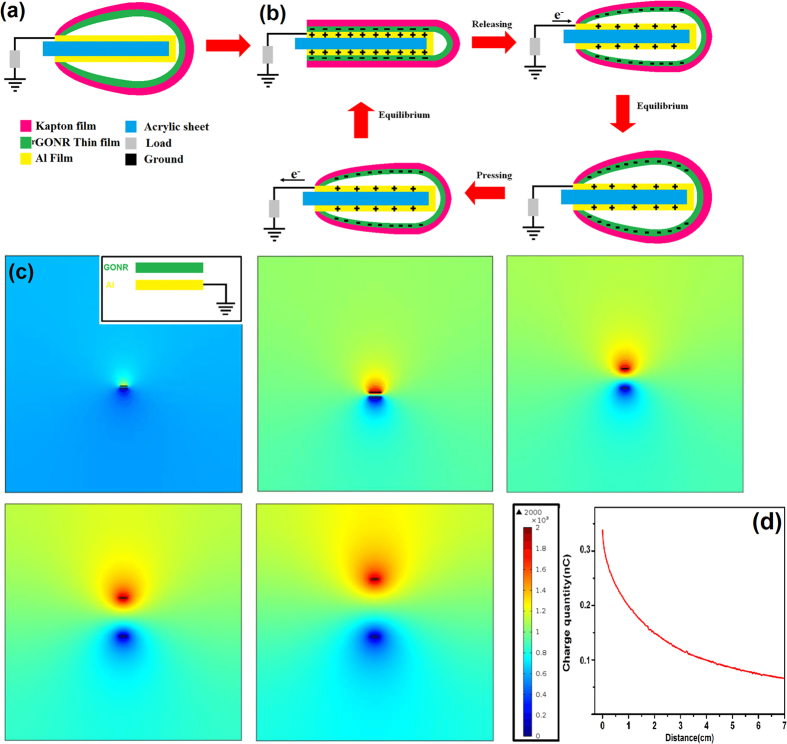
(**a**) Shows the schematic view of rGONRs/PVDF based single electrode TENG. (**b**) In contact and separation mode, how the electricity is produced by TENG. (**c**) COMSOL simulations for potential distribution at different gap distances 0.2, 3, 6, 9 and 12 cm, respectively between rGONRs/PVDF thin film and Al foil. The inset shows the model for the calculation. (**d**) Charges produced on the Al foil are decreasing by increasing the gap distance between rGONRs/PVDF film and Al foil.

**Figure 7:**
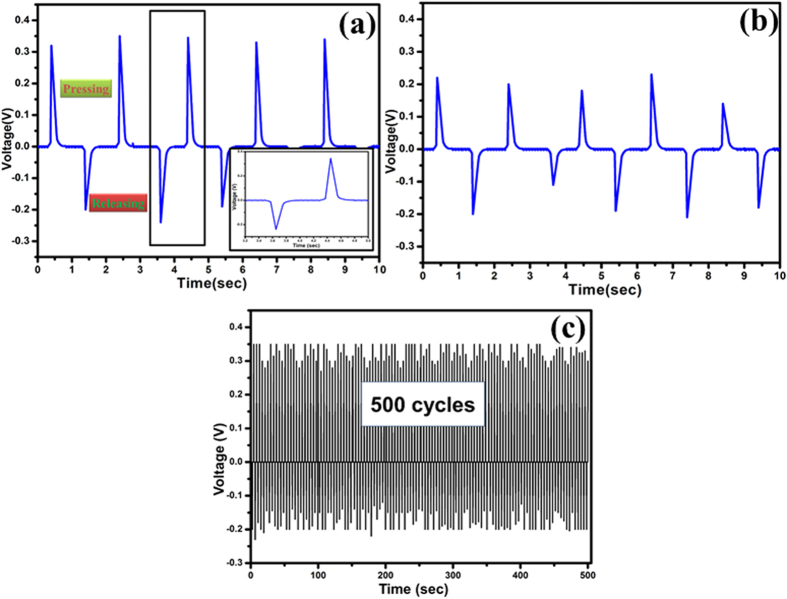
(**a**) Output voltage of the rGONRs/PVDF based triboelectric nanogenerator, (**b**) Output voltage of Kapton tape TENG (**c**) Shows that the rGONRs/PVDF based TENG was quite stable upto 500 cycles.

The triboelectric charge density could be increased by having the oxidized graphene nanoribbons on the surface, due to which the effective surface area has been enhanced. Figure 5d illustrates that the surface of the rGONRs/PVDF thin film was having the uniformly distributed nanoribbons structures. The working of the rGONRs/PVDF based TENG was performed by applying a mechanical force on the nanogenerator, therefore rGONRs/PVDF thin film and the Al foil can come in contact and released from each other. One of the electrode i.e. Al foil was connected to load resistance of 100 MΩ and was made ground. Once released, the rGONRs/PVDF thin film and Al would get apart from each other because of the elasticity stored in the kapton film, then come back in its original shape. The rGONRs/PVDF thin film surface was having negative surface electric potential can be upto 0.35 V by persistent contact-separation between the rGONRs/PVDF thin film and Al foil as illustrated in Fig. 6a. For measuring the voltage, TENG was connected to low noise voltage pre-amplifier. The output of the rGONRs/PVDF based single electrode TENG by applying the mechanical energy induced by finger touching was having highest voltage as 0.35 V. Moreover, we have removed rGONRs/PVDF thin film from Kapton. Now, Kapton is a contact surface in the fabricated TENG, corresponding electrical output voltage was found to be 0.16 V. When the compressive force was applied, the TENG would give positive voltage. While, compressive force was released it gave negative voltage, which is clearly depicted in Fig. 7.

The mechanism of the rGONRs/PVDF based TENG was schematically depicted as shown in Fig. 6. At the original state, no contact between Al foil and rGONRs/PVDF thin film, there was no electric output and hence no charge transfer across them. Once the Al foil and rGONRs/PVDF thin film surface came in contact with each other, electrons were injected from Al foil into rGONRs/PVDF thin film due to the presence of negative charge on itself. According to the triboelectric series, Al is having more propensity to loss electrons and hence according to electrostatic phenomena more triboelectrically positive than rGONRs/PVDF thin film^46^.

If the size of the rGONRs/PVDF thin film and Al foil was definite then the positive triboelectric charges, which were generated on the Al foil may drift to the ground through external load, giving electric field leakage. By releasing the mechanical force, TENG would instantly come back to its initial shape because of the flexibility present in the kapton tape. As the gap formed between the rGONRs/PVDF thin film and Al with definite size would be increasing, the triboelectric positive charge formed on the Al film was decreasing, which offers the flow of electrons from Al film to the ground. However, when the mechanical energy is applied again on the TENG to come in contact with each other i.e. Al and rGONRs/PVDF thin film, then induced triboelectric negative charges on the rGONRs/PVDF thin film would be increasing to balance the triboelectric positive charges on the Al foil and hence electrons would flow from ground into the Al foil. The full contact-separation cycle of electricity generation would be shown in Fig. 6b.

In order to further understand the working principle of the as prepared rGONRs/PVDF based single electrode TENG, numerical analysis was investigated through numerical simulation based on COMSOL Multiphysics. The model was constructed same as prepared experimentally, which based on Al foil and rGONRs/PVDF thin film with same size of 3 × 4.5 × 0.05 cm^3^. Figure 6d illustrates the calculations, which were performed and results in the electric potential distribution with the distance of 0.2, 3, 6, 9 and 12 cm, respectively between rGONRs/PVDF and Al foil. However, when the gap distance is increasing, the potential difference between the Al foil and rGONRs/PVDF thin film can increase upto 2000 V. As the gap distance between rGONRs/PVDF film and Al increases the amount of charges present on Al foil decreases, which indicates electrons were transferred from ground to Al foil as the gap distance is increasing. The principle behind the working of the as prepared TENG could be explained on the basis of transfer of charge among ground and Al foil, by varying the gap distance in the rGONRs/PVDF film and the Al foil due to the leakage field present at the edges of the thin films having definite size. The distance which was made between the rGONRs/PVDF thin film and Al foil must not be small while comparing it with the dimensions of the rGONRs/PVDF film or Al foil. If the gap distance between the rGONRs/PVDF thin film and Al foil is very less than the sizes of Al foil or the size of both the materials are approximately much large, then the charge transfer among the ground and Al might be very less. If this could be done, then the as prepared device might not work properly.

## Conclusions

In summary, we have synthesized the rGONRs through unzipping of CNTs. The charge storage and transfer capability of rGONRs/PVDF have been revealed by cyclic voltammetry. Therefore, we have designed single electrode TENG based on charge transfer between Al and rGONRs/PVDF thin film with finite size supported by kapton tape and modulating distance. In this approach, obtained AC voltage through finger touching convinced mechanical energy by TENG. The maximum output voltage would be achieved by TENG is 0.35 V. TENG could be quite stable upto 500 cycles and the energy generated by the TENG would be stored or can be directly used to operate portable electronics devices. This proposed work demonstrates the practicability of nanogenerator in which the mechanical energy is utilized and convert it into electrical energy.

## Materials and Methods

Multiwalled Carbon nanotubes (CNTs) were purchased from Hanwha Nanotech Co. (Republic of Korea). The length and outer diameter of MWCNTs were found to be 10–20 μm and 10–30 nm, respectively with average purities more than 95%. Poly (methyl methacrylate) (PMMA-IG 840) and Polyvinylidene fluoride (PVDF-6008/001 Solef) were purchased from Solvay, India and LG Chemical, respectively. Kapton film was purchased from Jay Industries, Mumbai, India; concentrated sulphuric acid (H_2_SO_4_) of 98% purity, Ether of 98% purity and dichloromethane (DCM) of 99% purity were purchased from Himedia laboratories Pvt. Ltd. Mumbai, India; potassium permanganate (KMnO_4_) of 99% purity was purchased from Rankem, RFCL Ltd. New Delhi, India; hydrogen peroxide (H_2_O_2_) of 30% purity was purchased from Loba Chemie Pvt. Ltd. Mumbai, India; dimethylformamide (DMF) of 99% purity was purchased from Avantor, Maharashtra, India and Al membrane was purchased from local market.

### Synthesis of reduced oxidized graphene nanoribbons

Reduced oxidized graphene nanoribbons (rGONRs) were synthesized by longitudinal unzipping of CNTs^47^. Generally, 150 mg of CNTs were steadily added in concentrated 150 ml of H_2_SO_4_ and stirrer for 24 hours, tailed by the addition of 750 mg of KMnO_4_ with stirring in oil bath, after this, a solution of 60 ml of 30% H_2_O_2_ and 300 ml deionized water (DI) was added. Achieved mixture was washed with 60 ml of 10% HCl solution, and then filtered. Ensuing product was dissolved in 50 ml of ethanol and followed by ultrasonication (750 W) for one hour. For exfoliation of obtained black dispersion mixture was washed several times with 50 ml diethyl ether. Resulting product was washed with distilled water to obtain neutral pH and desiccate in oven at 60 °C for 20 hrs without air. Moreover, synthesis process of oxidized graphene nanoribbons was shown in Fig. 1.

The thin film of rGONRs/PVDF was obtained by taking 97 wt% of rGONRs, which were dispersed in DMF through ultrasonicator for 2 hrs and say solution P1. On the other hand, 3 wt% of PVDF polymer as a binder was dissolved in organic solvent (DMF) solution at 150 °C and stirred for 30 minute and followed by the gradually addition of solution P1. Resulting mixture was used for preparation of thin film through drop casting technique and the prepared thin film was showed in Fig. 4c.

### Fabrication of Triboelectric nanogenerator

The schematic diagram of rGONRs/PVDF based triboelectric nanogenerator as shown in Fig. 4a. We are using PMMA sheet of 4.5 cm × 6 cm as substrate because of its good strength, light weight and acrylic material. The aluminum (Al) membrane of 2.5 cm × 4.5 cm was fixed on both sides of substrate which acts as electrode as well as triboelectric surface. A triboelectric thin film of rGONRs/PVDF (1.5 μm) was fixed on kapton film (125 μm). The kapton film was then bent in the arch shape around Al membrane and kapton tape was used to fix it on the PMMA sheet. Herein, TENG was having arch-shaped structure due to which both the films i.e. Al and rGONRs/PVDF thin film were having effective separation between them.

### Characterizations

The surface morphology of the rGONRs/PVDF thin film was examined by field emission Scanning Electron microscopy (FE-SEM). Before examining, a thin film of gold was sputtered on the sample surface. The unzipping of rGONRs were observed through by transmission electron microscopy (TEM) TECNAI G2 20 S-TWIN (FEI Netherlands), X-Ray diffraction (XRD) Bruker AXS Diffractometer D8 abvance with CuKα radiation, Atomic Force Microscope (AFM) NT-MDT NTEGRA, Fourier transform infrared spectroscopy (FTIR) by Perkin Elmer, Cyclic Voltammetry using EC epsilon, surface potential analysis Zetasizer Nano ZS and low noise voltage preamplifier (Keithley 6514 System Electrometer) was used to measure output performance of TENG.

## Additional Information

**How to cite this article**: Kaur, N. *et al*. Effective energy harvesting from a single electrode based triboelectric nanogenerator. *Sci. Rep.* **6**, 38835; doi: 10.1038/srep38835 (2016).

**Publisher's note:** Springer Nature remains neutral with regard to jurisdictional claims in published maps and institutional affiliations.

## Supplementary Material

Supplementary Information
