# Supplementary material for: Effective energy harvesting from a single electrode based triboelectric nanogenerator
Source: Sci Rep. 2016 Dec 13;6:38835. doi: 10.1038/srep38835 (PMC5153641; doi:10.1038/srep38835)
Supplement: Supplementary Information [file srep38835-s1.pdf]

Electronic Supplementary Information

Effective energy harvesting from a single electrode based triboelectric nanogenerator

Navjot Kaur[1], Jitendra Bahadur[1], Vinay Panwar[2], Pushpendra Singh[1], Keerti Rathi[1], Kaushik Pal[1,2,*]

[1] Centre of Nanotechnology, Indian Institute of Technology Roorkee, Roorkee, 247667, India.

[2] Department of Mechanical and Industrial Engineering, Indian Institute of Technology Roorkee, Roorkee, 247667, India.

*Corresponding Author: Kaushik Pal
E-mail: pl_kshk@yahoo.co.in

[*] Corresponding author
Tel no: +91-1332-284761; Fax: +91-1332-285665
E-mail address: pl_kshk@yahoo.co.in (K. Pal)

.

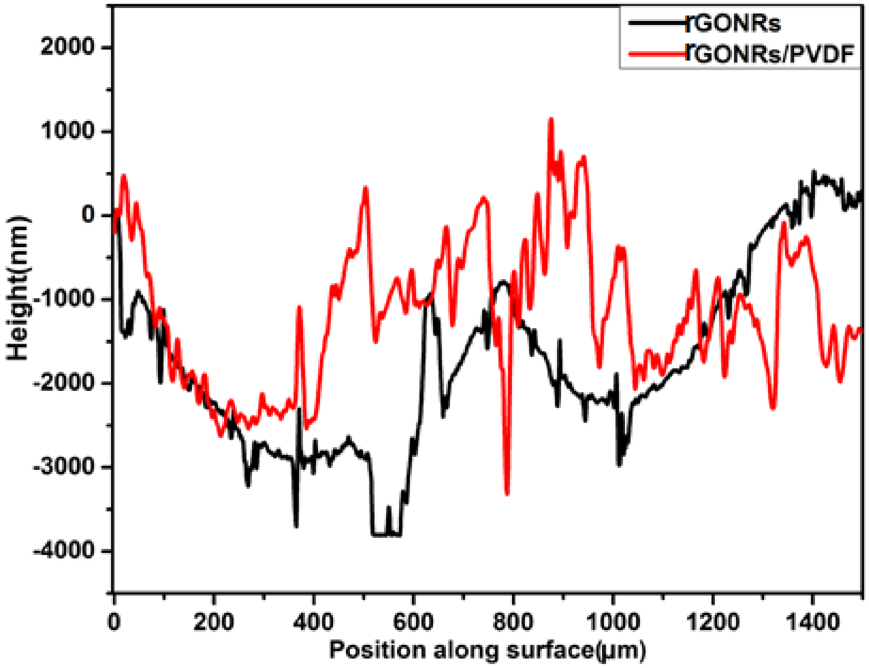

Figure S1: Surface Profilometery analysis has been performed to analyse the surface morphology of the rGONRs thin film and rGONRs/PVDF thin film.

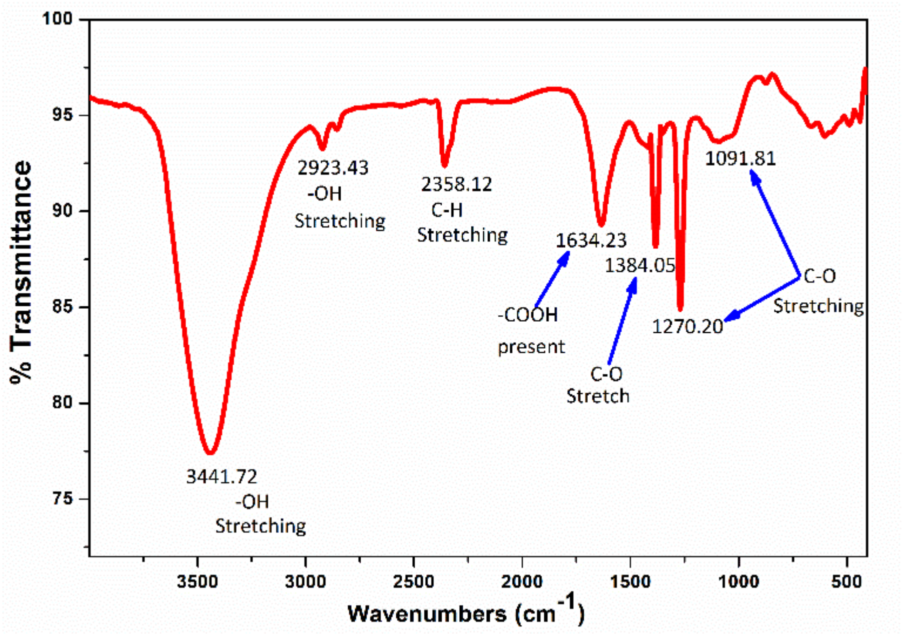

Figure S2: Fourier transform infrared spectroscopy (FTIR) of rGONRs.

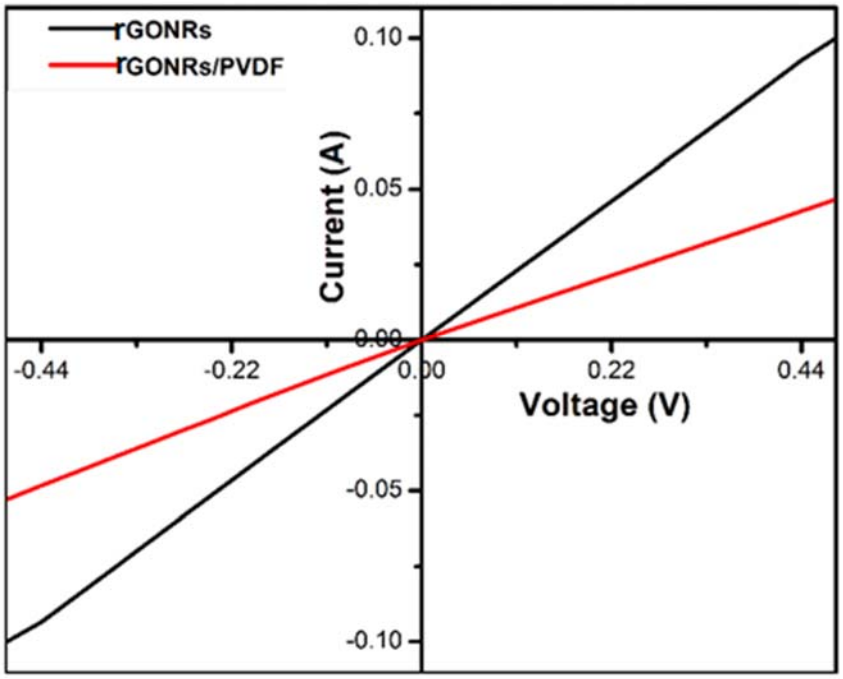

Figure S3: Two probe I-V characteristic measurement of the pristine rGONRs and rGONRs/PVDF composite.

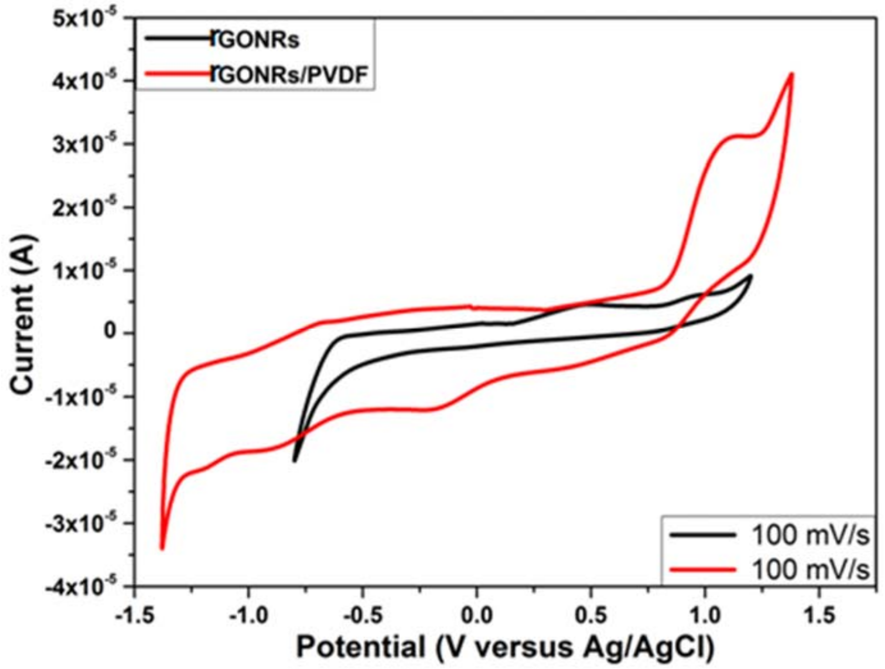

Figure S4: Cyclic voltammetry of the pristine rGONRs and rGONRs/PVDF composite.

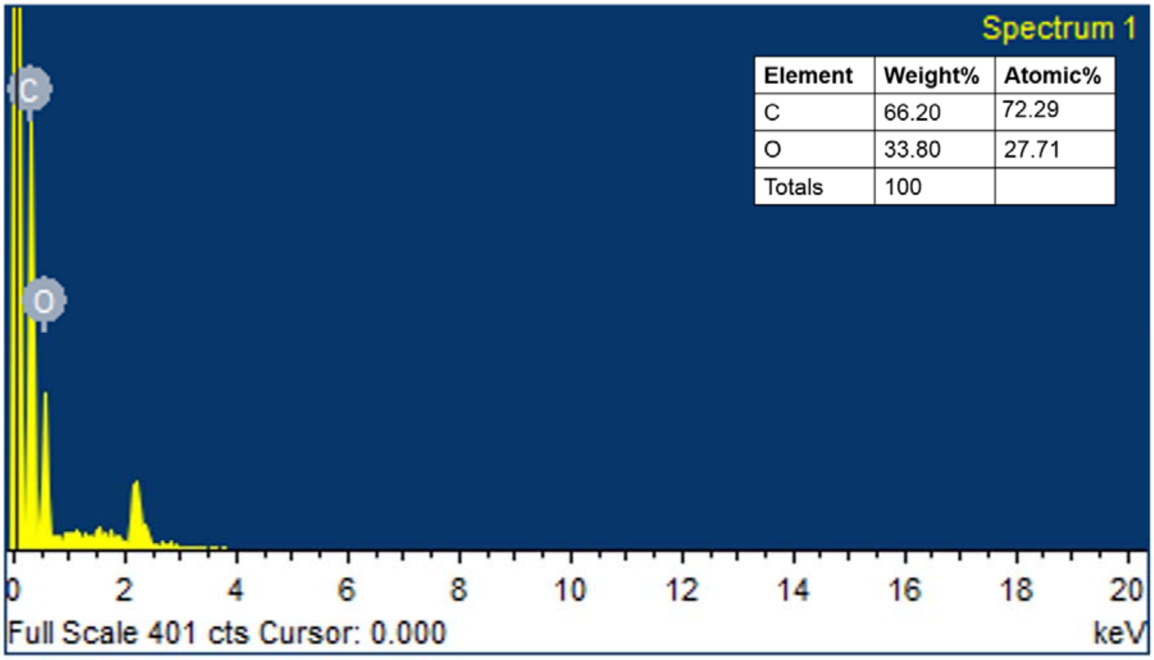

Element	Weight%	Atomic%
C	66.20	72.29
O	33.80	27.71
Totals	100	

Figure S5: Elemental analysis using energy dispersive X-ray analysis (EDAX) of the prepared rGONRs and the composition of C and O as shown above.

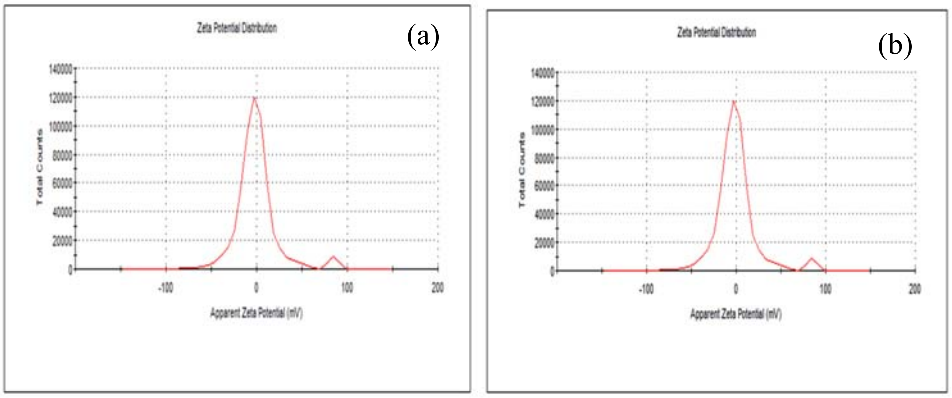

Figure S6: Surface charge analysis (a) rGONRs (b) rGONRs/PVDF composite.
